# Canonical correlation analysis on the association between pulmonary function and obesity in early-onset COPD: CT-based body composition analysis

**DOI:** 10.3389/fmed.2025.1633451

**Published:** 2025-09-22

**Authors:** Jiaru Shi, Tianye Li, Zhenghao Chen, Luoman Su, Qiongyan Wu, Hongjun Zhao, Chengshui Chen

**Affiliations:** ^1^Key Laboratory of Interventional Pulmonology of Zhejiang Province, Department of Pulmonary and Critical Care Medicine, The First Affiliated Hospital of Wenzhou Medical University, Wenzhou, China; ^2^The First Affiliated Hospital of Wenzhou Medical University, Wenzhou, China; ^3^Department of Pulmonary and Critical Care Medicine, Quzhou People’s Hospital, Zhejiang Province Engineering Research Center for Endoscope Instruments and Technology Development, The Quzhou Affiliated Hospital of Wenzhou Medical University, Quzhou, China

**Keywords:** chronic obstructive pulmonary disease, early-onset COPD, obesity, pulmonary function, body composition

## Abstract

**Background:**

While body composition impacts pulmonary function, the differential effects of visceral (VAT) vs. subcutaneous adipose tissue (SAT) in early-onset COPD remain unquantified.

**Objective:**

To elucidate the relationship between obesity patterns and pulmonary function in early-onset COPD versus non-COPD populations, focusing on body mass index (BMI), visceral adipose tissue (VAT), and subcutaneous adipose tissue (SAT) distribution.

**Methods:**

This retrospective cohort study analyzed 290 patients (144 early-onset COPD, 146 non-COPD) aged 20–50 years. Body composition (BMI, SAT, VAT) was quantified via CT at the L1 vertebral level. Pulmonary function was assessed by bronchodilator responsiveness testing (FEV₁/FVC, MEF₇₅, MEF₅₀, MEF₂₅). Canonical correlation analysis (CCA) was used to evaluate the multidimensional associations between body composition and pulmonary function.

**Results:**

Canonical correlation analysis revealed distinct multidimensional relationships between body composition and pulmonary function across study cohorts (*p* < 0.05). In the early-onset COPD cohort (N = 144), a statistically significant canonical variate (r = 0.383, *λ* = 0.172) demonstrated moderate association strength linking body composition (X_1_: BMI, SAT, VAT) with pulmonary function (Y_1_: FEV_1_, FVC, MEF_75_, MEF_25_). Conversely, the non-COPD group (N = 146) exhibited stronger canonical correlation (r = 0.537, *λ* = 0.405), with body composition (X_2_: BMI, VAT) associating with pulmonary function (Y_2_: FEV_1_, FVC).

**Conclusion:**

In summary, early-onset COPD patients with elevated BMI and VAT but reduced SAT exhibited improved pulmonary function across most parameters. This enhancement was not observed in MEF_50_ and MEF_25_. In contrast, the non-COPD cohort exhibits overall respiratory enhancement, as the cross-loading coefficient of MEF25—an indicator reflecting the weight of a variable in contributing to the canonical variate—is extremely small (0.05) and has a negligible impact on the overall association.

## 1 Introduction

Chronic obstructive pulmonary disease (COPD) is a heterogeneous lung condition characterized by chronic respiratory symptoms that cause persistent airflow obstruction, which leads to morbidity and mortality with an economic and social burden worldwide (1). The 2019 update of the Global Burden of Disease (GBD) ranked COPD 6th among all age groups in disability-adjusted life years (DALYs), significantly higher than in 1990. In 2019, COPD caused 3.28 million deaths and 74.43 million DALYs (2). The development of COPD is closely related to factors that contribute to persistent airflow limitation, and research evidence shows that smoking, occupational exposures, and indoor and outdoor air pollution are all associated with it. Conventional wisdom suggests that COPD is usually a disease of old age; however, the prevalence of COPD in young patients rises significantly with increased tobacco exposure (3). Patients with COPD who are younger than 50, referred to as early-onset COPD, have clinical characteristics that are not yet fully understood (1). According to the COPDGene cohort study, a significant proportion of COPD in young patients has a family history of respiratory disease or exposure to early life events. Prematurity, early life factors such as maternal smoking, bronchopulmonary dysplasia, severe childhood pneumonia, and malnutrition can impair normal lung function development, thereby preventing individuals from reaching peak lung function in early adulthood (4). Our study applied strict inclusion criteria and eliminated factors related to impaired lung function development mentioned above.

In recent years, the prevalence of obesity and being overweight among adults in China has been on the rise. A survey conducted among 15.8 million adults revealed that 34.8% were categorized as overweight, while 14.1% were classified as obese (5). Obesity significantly increases the risk of a variety of diseases and metabolic complications and is a frequent co-morbidity of COPD. There is an “obesity paradox” between obesity and COPD: several studies have found that while obesity is often associated with reduced lung function, obese patients with COPD tend to have lower morbidity and mortality rates compared to normal-weight patients (6, 7). Body mass index (BMI) is a straightforward and convenient method for categorizing the severity of obesity. However, it does not distinguish between central and peripheral obesity, nor does it consider the distribution patterns of visceral fat compared to subcutaneous fat. For this retrospective study, we used BMI, subcutaneous adipose tissue (SAT), and visceral adipose tissue (VAT) indexes to more accurately represent the degree of obesity.

While numerous studies report an inverse correlation between body composition and pulmonary function (8), others demonstrate a positive association linking BMI to pulmonary function (9–11). One study suggests that after multiple adjustments, VAT and SAT remained significantly negatively associated with FEV1 and FVC (12). In contrast, another study indicates that only VAT is negatively correlated with FEV1 and FVC (13). Most of the studies mentioned primarily focus on elderly COPD patients, leaving the nature and underlying mechanisms of the association between early-onset COPD and obesity unclear. To our knowledge, there has been no report on the association between pulmonary function and body composition in the early-onset COPD group. This study, which involves chest CT scans and bronchodilator reversibility tests, along with an in-depth examination of factors related to early-onset COPD and obesity, may uncover the underlying mechanisms of this association. The results recommend implementing strategic weight management through controlled augmentation of fat-free mass and subcutaneous adipose tissue reduction to optimize pulmonary function parameters in both healthy populations and COPD patients.

## 2 Methods

### 2.1 Study population

**Figure 1:**
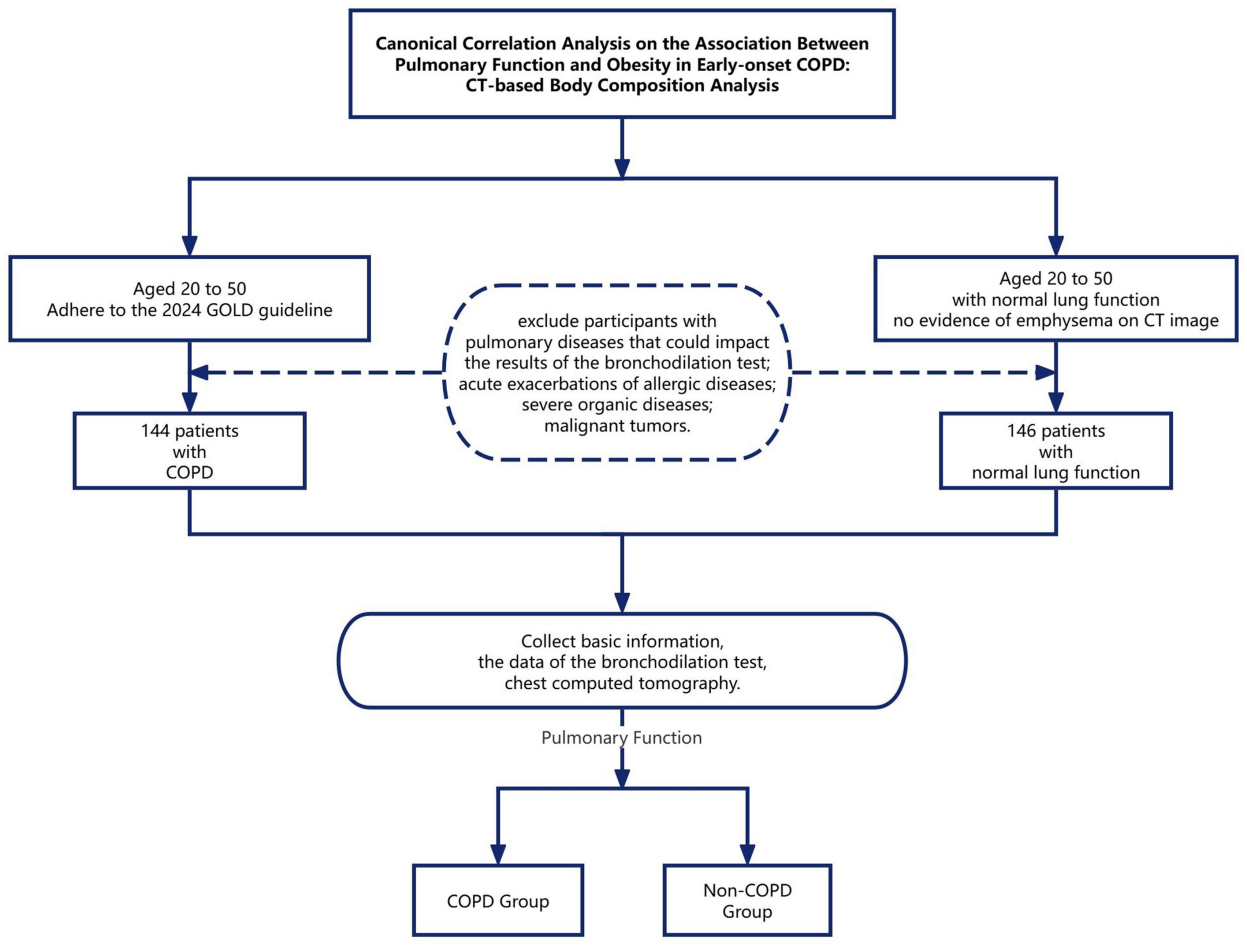
Canonical correlation analysis on the association between pulmonary function and obesity in early-onset COPD: CT-based body composition analysis.

This retrospective cohort study utilized data from patients aged 20–50 years treated at the First Affiliated Hospital of Wenzhou Medical University’s Department of Respiratory Medicine between January 2019 and December 2024. Patients demonstrating a post-bronchodilator forced expiratory volume in 1 s to forced vital capacity ratio (FEV₁/FVC) < 0.7 with negative bronchodilator reversibility (defined as ΔFEV₁ < 12% and < 200 mL) were classified into the early-onset COPD cohort. In contrast, patients with normal lung function and no evidence of emphysema or pulmonary bullae on CT images were categorized as the non-COPD group. Exclusion criteria comprised significant respiratory conditions (including asthma, pulmonary infections, tuberculosis, bronchiectasis, pneumoconiosis, or any other pulmonary disease potentially affecting bronchodilation test results), acute allergic or parasitic conditions (specifically acute exacerbations of allergic diseases or active parasitic infections), and severe systemic diseases (such as advanced heart failure, uremia, malignant tumors, or other severe organic diseases). Finally, 144 patients with the early-onset COPD group and 146 patients with the non-COPD group were included for further analysis (Figure 1).

### 2.2 Measurement

The study data included patients’ basic information, bronchodilator reversibility test results, chest computed tomography images, and measurements of subcutaneous adipose tissue (SAT) and visceral adipose tissue (VAT) obtained via CT.

Pulmonary function data included measurements taken before and after the inhalation of a bronchodilator (400 μg Ventolin). The parameters assessed were: Forced Expiratory Volume in the first second (FEV1); Forced Vital Capacity (FVC); The ratio of FEV1 to FVC (FEV1/FVC); Maximum Expiratory Flow at 75% of Forced Vital Capacity (MEF_75_); Maximum Expiratory Flow at 50% of Forced Vital Capacity (MEF_50_); Maximum Expiratory Flow at 25% of Forced Vital Capacity (MEF_25_). The volume-time curve (V-T curve) depicts the relationship between exhalation duration and volume change. Key parameters derived from this curve include Forced Vital Capacity (FVC), defined as the total volume exhaled as forcefully and completely as possible from full inspiration; Forced Expiratory Volume in the first second (FEV₁), which represents the expiratory volume during the initial second of FVC maneuver—demonstrating the highest reproducibility in pulmonary function testing, serving as a primary metric for bronchodilator and bronchoprovocation studies, and constituting the most widely applied parameter for assessing disease severity; and the FEV₁/FVC ratio, recognized as the gold-standard indicator for detecting airflow obstruction (14). The flow-volume curve (F-V curve) quantifies gas flow rates during maximal effort, deep, and rapid inspiratory or expiratory maneuvers. Key parameters extracted from this curve include maximum expiratory flow at 25, 50, and 75% of vital capacity (MEF₂₅, MEF₅₀, MEF₇₅), which serve as sensitive indicators of small airway function. The configuration and metric values of both curves are governed by the interdependent effects of expiratory effort, thoracopulmonary elastic recoil.

**Figure 2:**
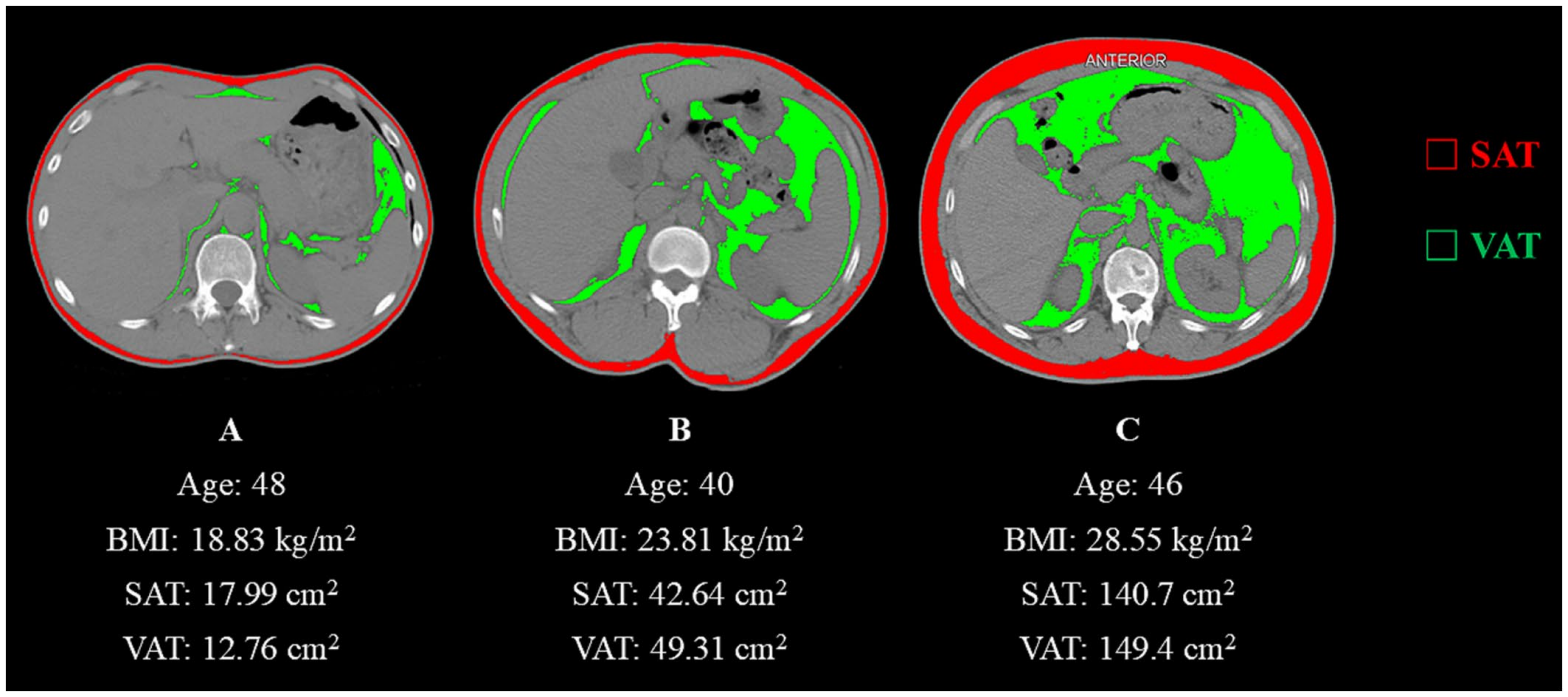
**(A)** Abdominal CT image with a Hounsfield unit-based color scale of adipose area in a 48-year-old man with a smoking history shows a lean physique. **(B)** Abdominal CT image with a Hounsfield unit-based color scale of adipose area in a 40-year-old man with a history of occupational paint exposure displays intermediate adiposity. **(C)** Abdominal CT image with a Hounsfield unit-based color scale of adipose area in a 40-year-old manifests marked obesity-related characteristics.

Instantaneous lung volume, and airway resistance on expiratory flow dynamics. Conventional quantification of SAT and VAT primarily relies on cross-sectional imaging at the umbilical level. Established modalities include magnetic resonance imaging (MRI), computed tomography (CT), dual-energy X-ray absorptiometry (DXA), and bioelectrical impedance analysis (BIA). MRI offers high accuracy without the use of ionizing radiation, but it is costly. DXA provides high precision for whole-body or regional body composition analysis, though its minimal radiation exposure limits routine clinical use (15). CT demonstrates superior precision to BIA and remains widely applied in clinical assessments (16). Thus, CT was selected as the tool for measuring body composition-related indices for this study. However, respiratory diseases are typically assessed using chest CT scans, while abdominal scans are infrequently utilized. In reviewing the relevant literature, Jung Hee Hong et al. found a strong correlation between whole-body composition and the L2-3 and L1 vertebral levels, with correlation coefficients of 0.90 and 0.89, respectively (17). This analysis was performed using DeepCatch, a fully automated deep learning software widely applied in clinical studies (18, 19). Therefore, the transverse section at the upper endplate of the individual L1 vertebra that the chest CT images could cover was selected as the image acquisition plane for this study. The chest CT images were processed using the SliceOmatic software version 5.0 (TomoVision, Montreal, Canada). In the CT images, the Hounsfield unit (HU) densities of muscles, blood vessels, intestines, adipose tissue, and bones vary significantly. Based on this principle, the software automatically identifies tissue discrepancies using previously validated Hounsfield unit (HU) density thresholds: −190 to −30 for subcutaneous adipose tissue (20) and −150 to −50 for visceral adipose tissue (21). This enables the removal of misclassified tissues with similar radiodensity while allowing manual incorporation of omitted adipose regions during screening. All participants were scanned with a 16-slice spiral CT system (uCT710 model, United Imaging Healthcare, Shanghai) using 5-mm-thick slices and 120-kV tube voltage. Image segmentation was performed by an experienced researcher unaware of clinical data, with subsequent verification conducted by a second investigator. The software automatically calculated the areas filled with different colors, using red to represent SAT and green to represent VAT. Figure 2 presents axial CT cross-sectional images of three males with distinct somatotypes, with quantitative delineations of SAT and VAT.

### 2.3 Statistical analysis

Continuous variables that conformed to normal distribution were expressed as mean ± standard deviation, and differences between groups were tested by an independent samples *t*-test. Continuous variables that did not conform to normal distribution were described as median (quartiles), and differences between groups were tested using non-parametric tests. Categorical variables were described as frequencies (percentages), and differences between groups were tested using the chi-square test. Canonical correlation analysis was used to analyze the correlation between body composition and pulmonary function. The sign of the standardized coefficient (indicating whether it is positive or negative) reveals the direction of influence that the raw variable has on the canonical variate. Additionally, the magnitude of the standardized coefficient reflects the strength of the variable’s impact on the canonical variate. A larger absolute value signifies a greater contribution of the raw variable to the canonical variate, meaning that this raw variable primarily determines the canonical variate. All statistical analyses were performed using IBM SPSS Statistics 27.0, with *p* < 0.05 deemed statistically significant.

## 3 Results

### 3.1 Analysis of baseline information

As shown in Table 1, a total of 290 participants were included in this study. There were 248 men (85.52%) and 42 women (14.48%), with a median age of 47 years. The cohort comprised 144 patients with early-onset COPD and 146 patients with normal pulmonary function. Baseline characteristics, including age, gender, data source, height, weight, BMI, SAT, and VAT, showed no statistically significant differences between the groups (*p* > 0.05). However, statistically significant differences were observed in smoking status (*p* < 0.001) and all pulmonary function parameters (FEV₁, FVC, FEV₁/FVC ratio, MEF₇₅, MEF₅₀, MEF₂₅, MMEF; *p* < 0.001), consistent with the clinical distinction between COPD and non-COPD populations.

**Table 1:** Values of clinical objective indicators in patients of the two groups.

Variables	Total (*n* = 290)	Early-onset COPD	Non-COPD	*p* value
*N* (%)	290	144 (49.66%)	146 (50.34%)	
Age, years	47 (44,48)	47 (44.25,49)	46 (44,48)	0.064
Gender, *n*(%)				0.341
Male	248 (85.52%)	126 (87.50%)	122 (83.60%)	
Female	42 (14.48%)	18 (12.50%)	24 (16.40%)	
Data sources, *n*(%)				0.246
Outpatients	288 (99.31%)	142 (98.60%)	146 (100.00%)	
Hospitalized patients	2 (0.69%)	2 (1.40%)	0	
Smoking, *n*(%)				<0.001
Yes	106 (36.55%)	72 (50.00%)	34 (23.30%)	
No	21 (7.24%)	9 (6.30%)	12 (8.20%)	
Unknown	163 (56.21%)	63 (43.80%)	100 (68.50%)	
Occupation exposure	16	11	5	-
Height (cm)	169 (165,172)	169 (165,170.75)	169 (165,172)	0.684
Weight (Kg)	65.60 (60,74)	66.04 ± 11.38	67.00 ± 9.73	0.44
FEV_1_	3.19 (2.53,3.75)	2.55 ± 0.83	3.59 ± 0.04	<0.001
FVC	4.09 (3.48,4.55)	3.82 ± 0.94	4.09 ± 0.05	<0.001
MEF_75_	5.16 (3.90,8.50)	3.90 ± 1.67	8.47 (7.52,9.32)	<0.001
MEF_50_	3.28 (1.79,5.26)	1.79 (1.18,2.38)	5.24 (4.35,6.07)	<0.001
MEF_25_	1.08 (0.58,1.88)	0.58 (0.39,0.71)	1.88 (1.54,2.38)	<0.001
BMI (Kg/m^2^)	23.81 (21.50,25.71)	23.30 ± 3.28	23.70 ± 2.68	0.263
SAT (cm^2^)	57.52 (37.92,81.71)	55.03 (32.03,81.16)	63.32 (39.43,82.60)	0.137
VAT (cm^2^)	60.05 (30.39,108.73)	55.18 (28.02,108.35)	62.31 (32.88,109.93)	0.365

FEV_1_, forced expiratory volume in the first second; FVC, forced vital capacity; FEV_1_/FVC, the ratio of FEV1 to FVC; MEF_75_, maximum expiratory flow at 75% of forced vital capacity; MEF_50_, maximum expiratory flow at 50% of forced vital capacity; MEF_25_, maximum expiratory flow at 25% of forced vital capacity; BMI, body mass index; SAT, subcutaneous adipose tissue; VAT, visceral adipose tissue.

In the early-onset COPD group, occupational exposure information, including exposure type and duration, was collected from 11 individuals through review of clinician-documented medical records. Their exposures included paint (4), electroplating and welding (3), clothing (1), automobile repair (1), metal (1), and chemicals (1). In the non-COPD group, information was gathered from 5 individuals, whose exposures included dust (2), furniture factory (1), and clothing factory (1).

### 3.2 Canonical correlation analysis of body composition and pulmonary function

#### 3.2.1 Spearman’s correlation

Table 2 presents the correlation coefficient matrix among the three domains of body composition and the five components of pulmonary function testing parameters in patients from the two groups. In the early-onset COPD group, most correlations were weak. The strongest observed association—though still weak—was between BMI and MEF_75_ (r = 0.285, *p* = 0.001). Conversely, in the Non-COPD group, only SAT showed significant correlations with lung function parameters, demonstrating a moderate positive correlation with FEV_1_ (r = 0.388, *p* = 0.001).

**Table 2:** Spearman’s correlation between body composition and pulmonary function testing parameters from two groups.

Body composition parameters			FEV_1_	FVC	MEF_75_	MEF_50_	MEF_25_
COPD group	BMI	Spearman’s correlation coefficient	0.277	0.202	0.285	0.283	0.142
		*P* value	0.001	0.015	0.001	0.001	0.089
	SAT	Spearman’s correlation coefficient	0.092	−0.024	0.089	0.139	0.015
		*P* value	0.275	0.777	0.29	0.097	0.86
	VAT	Spearman’s correlation coefficient	0.254	0.141	0.253	0.242	0.08
		*P* value	0.002	0.091	0.002	0.003	0.341
Non-COPD group	BMI	Spearman’s correlation coefficient	0.025	0.067	0.074	0.018	−0.106
		*P* value	0.761	0.421	0.375	0.828	0.201
	SAT	Spearman’s correlation coefficient	−0.388	−0.364	−0.038	−0.228	−0.233
		*P* value	<0.001	<0.001	0.652	0.006	0.005
	VAT	Spearman’s correlation coefficient	0.087	0.09	0.092	0.12	0.048
		*P* value	0.298	0.279	0.27	0.148	0.568

#### 3.2.2 Canonical correlation

To understand the relationship between the two groups of indicators, we conducted a canonical correlation analysis for data description, as shown in Table 3. We considered the body composition indices as a set of composite variables (X), which included BMI, SAT, and VAT, and constructed linear equations based on these variables. Meanwhile, the pulmonary function data were treated as another set of composite variables (Y), with linear equations formulated from the measurements of FEV_1_, FVC, MEF_75_, MEF_50_, and MEF_25_ after the inhalation of bronchodilators.

**Table 3:** Outcomes of canonical correlation analysis.

Group		Canonical correlation (r)	λ	Wilks’	F	*p* value
COPD group	1	0.383	0.172	0.777	2.393	0.003
	2	0.288	0.090	0.911	1.631	0.116
	3	0.081	0.007	0.993	0.306	0.821
Non-COPD group	1	0.537	0.405	0.676	3.877	<0.001
	2	0.198	0.041	0.95	1.631	0.511
	3	0.106	0.011	0.989	0.531	0.662

As indicated in Table 3, the canonical correlation coefficient for the first pair of canonical variates in the early-onset COPD group was 0.383, accompanied by an eigenvalue of 0.172 (*p*-value of 0.003 from the likelihood ratio test). In contrast, the canonical correlation coefficient for the first pair of canonical variates in the non-COPD group was 0.537, with an eigenvalue of 0.405 (*p*-value of less than 0.001 from the likelihood ratio test). The conclusion indicates a relationship between body composition and pulmonary function in both groups.

**Figure 3:**
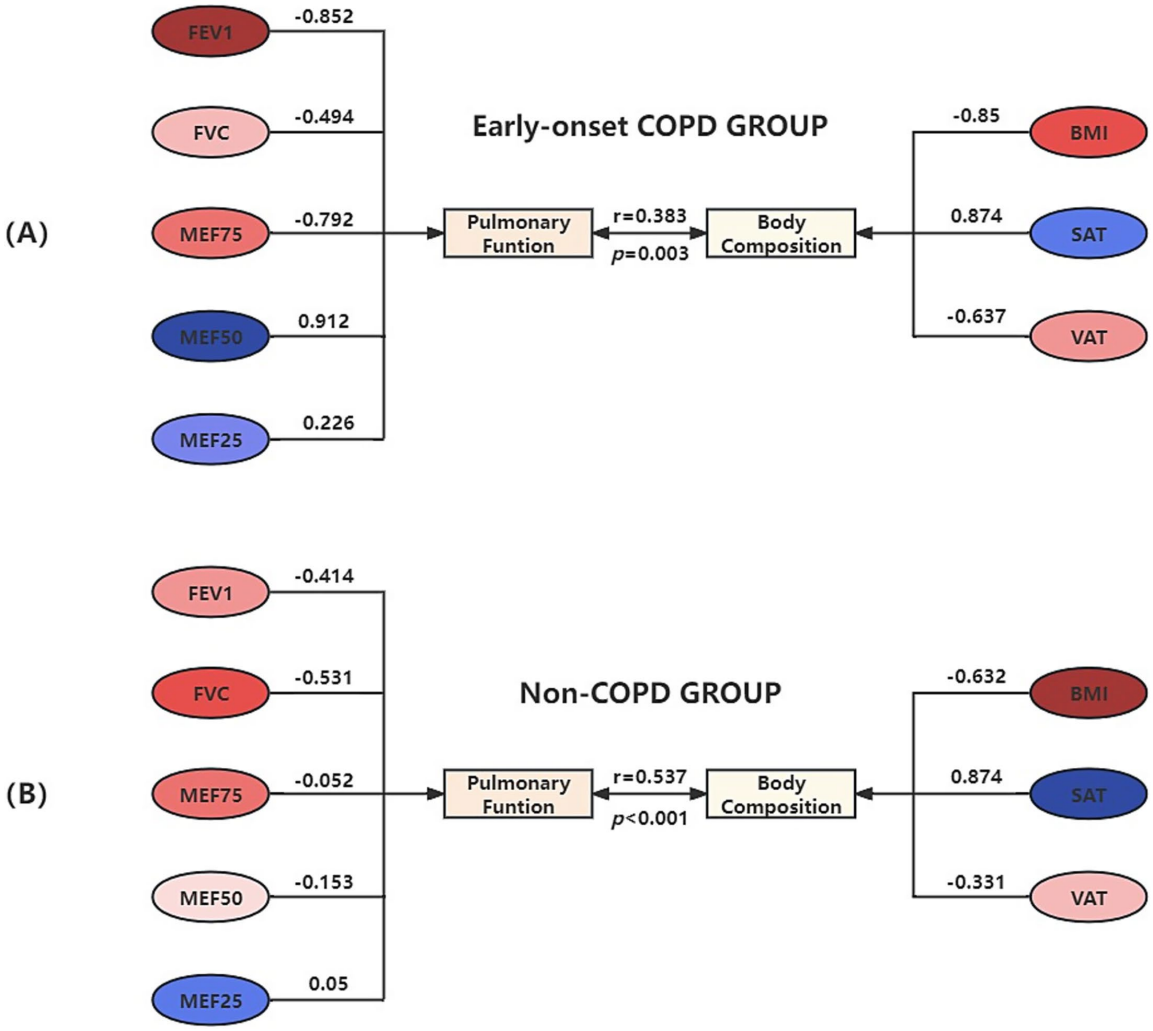
Canonical correlation structure chart of body composition and pulmonary function in the early-onset COPD Group **(A)** and Non-COPD Group **(B)**.

Figure 3 presents the result of the canonical correlation between body composition and pulmonary function. If the variables in the two groups are the same color (red vs. blue), it indicates a positive correlation between them. Otherwise, they are negatively correlated. The gradation of color represents the weight of the variable to the canonical variate.

In the early-onset COPD group, X_1_ represented the first pair of canonical variates for body composition: X_1_ = −0.85BMI + 0.874SAT − 0.637VAT; Y_1_ represented the first pair of canonical variates for pulmonary function: Y_1_ = −0.852FEV_1_-0.494FVC-0.792MEF_75_ + 0.912MEF_50_ + 0.226MEF_25_. The linear combination of body composition factors X_1_, mainly determined by BMI, SAT, and VAT, indicates an association with pulmonary function; the linear combination of pulmonary function factors Y_1_, mainly determined by FEV_1_, FVC, MEF_75_, and MEF_25_, indicates an association with body composition. BMI and VAT showed a positive correlation with FEV_1_, FVC, and MEF_75_, indicating that higher levels of BMI and VAT were associated with improved pulmonary function in these measures. Conversely, there was a negative correlation with MEF_50_ and MEF_25_, meaning that as BMI and VAT increased, these measurements of pulmonary function decreased. In contrast, the relationship with SAT was the opposite.

In the non-COPD group, X_2_ represented the first pair of canonical variates for body composition: X_2_ = −0.632BMI + 0.874SAT − 0.331VAT; Y_2_ represented the first pair of canonical variates for pulmonary function: Y_2_ = −0.414FEV_1_–0.531FVC − 0.052MEF_75_ − 0.153MEF_50_ + 0.05MEF_25_. The linear combination of body composition X_2_, mainly determined by BMI and VAT, indicates an association with pulmonary function; the linear combination of pulmonary function factors Y_2_, mainly determined by FEV_1_ and FVC, indicates an association with body composition. Given the small cross-loading of the MEF_25_, it can be assumed that both BMI and VAT positively correlate with pulmonary function, while SAT is negatively correlated.

## 4 Discussion

In this study, significant positive correlations between body composition and pulmonary function were observed in both cohorts (early-onset COPD: r = 0.383, *p* < 0.05; non-COPD: r = 0.573, *p* < 0.05).

### 4.1 In the early-onset COPD group

Elevated BMI demonstrates positive correlations with increased FEV_1_, FVC, and MEF_75_ values, while showing negative correlations with MEF_50_ and MEF_25_ levels. Extensive evidence indicates that underweight status in COPD patients correlates with adverse clinical outcomes, encompassing elevated mortality rates, heightened exacerbation frequency, and impaired pulmonary performance (22–24). Supporting this observation, a large prospective cohort study in Japan observed that 45,837 male residents identified low BMI and progressive weight loss as significant predictors of COPD mortality (25). A meta-analysis of studies involving over 27,000 participants from clinical trials has shown the potential protective effects of higher BMI against lung function decline (24). K. B. H. Lam et al. (26) analyzed data from 7,358 individuals aged 50 and older. They found that when adjusted for BMI, airflow limitation was strongly associated with central obesity, which is a component of metabolic syndrome. These collective findings align with our current investigation, proposing that moderate BMI elevation might reflect favorable nutritional profiles, enhanced fat-free mass index (FFMI) (27), superior respiratory muscle capacity, and sustained ventilatory efficiency. The paradoxical reduction in small airway parameters (MEF_50_, MEF_25_) could potentially stem from BMI-related abdominal adiposity, which raises abdominal pressure, pushes the diaphragm upward, and ultimately reduces pulmonary hyperinflation and pulmonary volume (28).

Elevated VAT levels are associated with positive correlations for FEV_1_, FVC, and MEF_75_, alongside negative correlations with MEF_50_ and MEF_25_. Accumulating evidence identifies adipose tissue as an endocrine organ capable of inducing low-grade systemic inflammation through adipocytokine secretion, including interleukin-6 (IL-6), plasminogen activator inhibitor-1 (PAI-1), and leptin. A longitudinal cohort study involving 3,075 elderly participants, aged 70–79 years, revealed that VAT accumulation positively correlates with systemic inflammatory biomarkers, wherein IL-6 demonstrated particularly strong predictive value for mortality in COPD populations (29). Complementary findings by Bunk et al. demonstrated significant associations between emphysema progression, bronchial wall thickening, and thoracic fat-muscle composition in smoking-related COPD, suggesting VAT-driven chronic inflammation may potentiate airway remodeling (30). The observed discrepancy between our findings and established paradigms may stem from the investigation of specific population subgroups, utilization of non-conventional CT planes for visceral adipose tissue (VAT) quantification, and the absence of corroborative evidence in existing literature. We propose a novel mechanistic hypothesis: VAT expansion stimulates adiponectin (APN) overproduction, which activates AMP-activated protein kinase (AMPK), peroxisome proliferator-activated receptors (PPARs), extracellular signal-regulated kinases (ERK), and protein kinase B (AKT) through adiponectin receptor (AdipoR)-mediated signaling in pulmonary cells. This cascade may ultimately attenuate inflammatory responses and enhance airway compliance (31).

When SAT is elevated, negative correlations occur for FEV_1_, FVC, and MEF_75_, while positive correlations are observed for MEF_50_ and MEF_25_. This pattern may occur because excess subcutaneous fat adds weight and thickness to the chest wall, which can limit the expansion and contraction of the thoracic cavity, ultimately negatively impacting respiratory function. Clinical studies have demonstrated that obese individuals, characterized by excessive subcutaneous adipose tissue accumulation, exhibit reduced end-expiratory lung volumes (EELV). This physiological alteration necessitates elevated energy expenditure during respiratory efforts, thereby augmenting the respiratory load (32). Furthermore, emerging evidence suggests that subcutaneous adiposity may impair airway dynamics via pro-inflammatory mechanisms that compromise airway patency, exacerbating disease severity and hospitalization risk in chronic obstructive pulmonary disease populations. Nevertheless, current evidence remains inconclusive regarding sustained elevations in MEF_50_ and MEF_25_.

### 4.2 In the non-COPD group

Elevated BMI demonstrates positive associations with increased FEV_1_, FVC, MEF_75_, MEF_50_, and MMEF values, while showing a negative correlation MEF_25_. VAT accumulation mirrors BMI’s pattern, positively associating with all spirometric parameters except MEF_25_. Conversely, heightened subcutaneous adipose tissue (SAT) levels exhibit negative correlations with FEV_1_, FVC, MEF_75_, MEF_50_, but show a paradoxical elevation in MEF_25_. Given the minimal cross-loading coefficient (0.05) observed for MEF_25_, these findings collectively suggest enhanced pulmonary function under conditions of increased BMI and VAT, accompanied by decreased SAT.

This pattern aligns with a large-scale Korean cross-sectional study, reporting higher pulmonary function parameters in obese versus underweight individuals. Lower BMI tended to decrease pulmonary function parameters such as FEV_1_, predicted FEV_1_ (%), FVC, predicted FVC, and PEF (*p* < 0.001) (33), consistent with our observed BMI-pulmonary function correlations. Notably, recent investigations into adipose distribution patterns among healthy young adults revealed inverse correlations between lung function measures and both SAT/VAT (r < 0; *p* < 0.05) (34). However, the differential impacts of SAT versus VAT on pulmonary mechanics remain underexplored, with no comparable investigations reported in the current literature.

### 4.3 Smoking

Smoking status is obtained from medical records created by physicians during patient visits; however, documentation is sometimes lacking in detail. We categorized smoking status into three groups: smokers, non-smokers, and missing data. A total of 163 patients were found to have missing data regarding their smoking status, including 63 patients from the early-onset COPD group and 100 from the non-COPD group. In Table 1, there is a statistically significant difference in smoking status between these two groups. Smoking may be an independent risk factor for chronic obstructive pulmonary disease, but there are other potential explanations for the differences observed between COPD patients and those with normal lung function. One possibility is that doctors paid more attention to the smoking status of patients diagnosed with COPD, leading to more complete and accurate smoking histories in this group. This could introduce information bias. Additionally, patients diagnosed with COPD may have chosen to quit smoking voluntarily, while those with normal lung function did not experience the same pressure. Consequently, the observed differences in smoking prevalence between the two groups might reflect behavioral changes after the diagnosis rather than differences in their initial smoking exposure.

## 5 Conclusion

In summary, this study proposes that under conditions of elevated BMI and VAT with concomitant reduction in SAT: (1) The early-onset COPD patients demonstrate improved pulmonary function parameters except for MEF50 and MEF25; (2) The non-COPD cohort exhibits overall respiratory enhancement, supported by the minimal cross-loading coefficient (0.05) for MEF₂₅. These findings highlight three key implications for future clinical practice. First, routine quantification of VAT and SAT could refine risk assessment beyond BMI alone. For instance, pulmonary CT enables early identification of high-risk patients needing intervention. Second, clinical guidelines should incorporate precision weight management protocols including targeted SAT reduction through exercise or nutrition interventions to mitigate thoracic restriction, VAT-preserving strategies to maintain anti-inflammatory adipokine production, and fat-free mass augmentation to support respiratory musculature. Finally, serial CT adiposity measurements could enable therapeutic monitoring by tracking the treatment efficacy of nutritional and pharmacological interventions. Therefore, we advocate integrating CT-derived body composition metrics into pulmonary health assessments to enable personalized interventions for maintaining optimal respiratory function in both COPD and general populations.

Nevertheless, this investigation has several methodological constraints. Although rigorous inclusion/exclusion criteria yielded a final cohort of 290 participants, subsequent subgroup stratification diminished sample sizes, inevitably amplifying sampling bias and potentially compromising the reliability and generalizability of findings. The standardized selection of the L1 superior endplate axial plane for imaging acquisition, while evidence-based, may not adequately account for body composition heterogeneity in abdominal obesity populations, thereby limiting the validity and applicability of results when extrapolated to this specific demographic. Critically, the omission of fat-free mass (FFM) quantification represents a significant limitation. Our findings of improved pulmonary function associated with elevated VAT may partially reflect FFM augmentation rather than solely adiposity effects. Furthermore, the retrospective design inherently restricted data completeness due to reliance on clinician-documented medical records.

## Data Availability

The raw data supporting the conclusions of this article will be made available by the authors, without undue reservation.
